# Association between serum vitamin D levels and the risk of diabetic retinopathy: a meta-analysis

**DOI:** 10.3389/fmed.2026.1771156

**Published:** 2026-04-01

**Authors:** Yi Lan, Xinyi Ke

**Affiliations:** Department of Ophthalmology, The Traditional Chinese Medicine Hospital of Longquanyi, Chengdu, Sichuan, China

**Keywords:** 25-hydroxyvitamin D, diabetic retinopathy, meta-analysis, observational studies, risk factor, vitamin D

## Abstract

**Aim:**

This meta-analysis was conducted to quantitatively synthesize existing evidence on the relationship between serum 25-hydroxyvitamin D [25(OH)D] levels and diabetic retinopathy (DR).

**Methods:**

A systematic search of PubMed, Embase, the Web of Science, and the Cochrane Library was performed for obtaining observational studies published between January 2015 and February 2026. The included studies reported adjusted effect estimates for the association between 25(OH)D levels and DR. A random-effects model was used to calculate pooled odds ratios (ORs) with 95% confidence intervals (CIs). Subgroup analysis was performed to explore the sources of heterogeneity based on ethnicity, diabetes type, study design, and vitamin D assay method. A sensitivity analysis was conducted to evaluate the robustness of the pooled results. Publication bias was assessed using Begg’s funnel plot and Egger’s linear regression test.

**Results:**

This meta-analysis included 16 relevant studies, and the pooled results showed that low serum vitamin D levels were associated with a higher likelihood of developing DR (OR = 1.87, 95% CI: 1.66–2.11). Subgroup analyses revealed stronger effect sizes in Asian populations (OR = 1.97, 95% CI: 1.63–2.38) and type 1 diabetes mellitus (T1DM) patients (OR = 2.15, 95% CI: 1.70–2.72). The sensitivity analysis confirmed the robustness of the findings, and publication bias was not observed.

**Conclusion:**

Low vitamin D levels were associated with higher odds of developing DR, and this association was particularly evident in Asian individuals and T1DM patients. These findings suggest the importance of monitoring vitamin D levels in the management of DR.

## 1 Introduction

Diabetic retinopathy (DR) is one of the most frequent and severe microvascular complications of diabetes mellitus and a leading cause of preventable visual impairment and blindness among working-age adults worldwide (1). According to recent estimates from the International Diabetes Federation (IDF), more than 530 million individuals are living with diabetes globally, with approximately one-third of these patients expected to develop some degree of DR during their disease course (1). The escalating prevalence of diabetes worldwide further exacerbates the clinical and public health burden imposed by this ocular complication (1).

The pathogenesis of DR is multifactorial and complex. Chronic hyperglycemia is recognized as a key initiating factor that triggers a cascade of pathological processes, including oxidative stress, protein kinase C (PKC) activation, and advanced glycation end-product (AGE) accumulation, ultimately leading to blood-retinal barrier dysfunction, retinal ischemia, and pathological angiogenesis. However, significant heterogeneity in DR progression has been observed among patients with comparable glycemic control (2). This clinical observation suggests the involvement of additional factors beyond glucose metabolism.

Vitamin D, a secosteroid hormone, has attracted increasing interest for its extraskeletal functions in recent years (3). Beyond its classical role in mineral homeostasis, vitamin D demonstrates pleiotropic effects, including immunomodulatory, anti-inflammatory, anti-fibrotic, and anti-angiogenic properties (4). Preclinical studies suggest that vitamin D can suppress the NF-κB inflammatory pathway, thereby reducing the production of pro-inflammatory cytokines such as TNF-*α* and IL-6 (5). In addition, vitamin D improves endothelial function by enhancing endothelial nitric oxide synthase (eNOS) activity and exerts inhibitory effects on the renin–angiotensin system (RAS) and the expression of vascular endothelial growth factor (VEGF), which is a critical mediator responsible for vascular leakage and neovascularization in DR (6). These diverse biological actions provide a strong rationale for investigating the association between vitamin D levels and the risk of DR.

Numerous clinical studies have explored the association between vitamin D levels and DR; however, the findings are inconsistent (7–9). Chen et al. (7) and Hung et al. (8) reported that vitamin D deficiency is independently associated with an increased risk of DR, while Alam et al. (9) found no association between vitamin D and DR. These discrepancies may be attributable to differences in study design, participant ethnicity, sample size, vitamin D measurement, adjustment for confounders (e.g., diabetes duration and renal function), and DR diagnostic criteria (10).

Due to the limitations and conflicting findings of individual observational studies, a systematic synthesis of the available evidence is warranted. Therefore, a meta-analysis is conducted to comprehensively identify and integrate relevant studies in order to provide a robust quantitative estimate of the association between vitamin D levels and the risk of DR. Furthermore, we sought to explore the potential sources of heterogeneity across studies through subgroup analyses. This meta-analysis may provide important insights for clinical guidance and public health policy-making regarding DR.

## 2 Methods

### 2.1 Literature search strategy

This meta-analysis was performed following the Preferred Reporting Items for Systematic Reviews and Meta-Analyses (PRISMA) guidelines (11). Electronic databases, including PubMed, Embase, the Web of Science, and the Cochrane Library, were systematically searched. The initial search was restricted to studies published between 1 January 2015 and 31 December 2024, with updated searches conducted on 24 February 2026. The search strategy is shown in Supplementary file 1. To minimize the omission of eligible studies, the reference lists of all included studies and relevant review articles were manually screened. The initial search imposed no language restrictions. However, for publications in languages other than Chinese or English, only the titles and abstracts were screened, with translation tools used as needed for preliminary assessments. Two independent reviewers performed study selection, and discrepancies were resolved through consensus or consulting a third reviewer. Inter-rater reliability was evaluated using the kappa statistic, and the kappa value was 0.89, indicating excellent agreement between the reviewers.

### 2.2 Literature inclusion and exclusion criteria

The inclusion criteria were defined *a priori* based on the PICOS framework: (P) Population: Individuals diagnosed with type 1 or type 2 diabetes mellitus, without restrictions on ethnicity, gender, or age. (I/E) Exposure: Serum or plasma 25-hydroxyvitamin D [25(OH)D] levels, measured using standardized methods (e.g., immunoassays and chromatography). The primary exposure was defined as vitamin D deficiency or insufficiency (commonly indicated by serum 25(OH)D levels <20 ng/mL or 50 nmol/L), compared to vitamin D sufficiency. (C) Comparison: Individuals with the lowest vitamin D levels versus those with the highest levels, or individuals with deficiency/insufficiency versus those with sufficiency. (O) Outcome: DR, diagnosed using standardized ophthalmological examinations such as fundus photography (Early Treatment Diabetic Retinopathy Study (ETDRS) or equivalent grading standards), fluorescein angiography, or clinical assessments conducted by an ophthalmologist. (S) Study design: Observational studies, including case–control, cross-sectional, and cohort designs.

The exclusion criteria were as follows: (1) non-original studies (e.g., reviews, meta-analyses, conference abstracts, and editorials) and preclinical studies (animal or cellular experiments); (2) articles with unavailable full texts or insufficient data for extraction or conversion of effect sizes; (3) studies involving patients with gestational diabetes or secondary diabetes; and (4) duplicate publications, with only the most comprehensive or recent studies retained in cases of duplication.

### 2.3 Data extraction

Data extraction was performed independently by two trained researchers, and the following data were extracted: First author, year of publication, country or region where the study was conducted, study design (case–control, cross-sectional, or cohort), type of diabetes (T1DM or T2DM), sample size, number of DR cases and total participants, vitamin D measurement method [e.g., chemiluminescent immunoassay (CLIA), liquid chromatography–tandem mass spectrometry (LC–MS/MS), or high-performance liquid chromatography (HPLC)], specific criteria or procedure used for DR diagnosis, and effect size data [fully adjusted odds ratios (ORs), hazard ratios (HRs), or relative risks (RRs) with corresponding 95% confidence intervals (CIs) from the multivariate model with the highest degree of adjustment for confounders].

### 2.4 Quality assessment

Given that all included studies were observational, the Newcastle–Ottawa scale (NOS) was used to evaluate methodological quality (12). The NOS uses a star rating system (maximum of nine stars) across three domains: Selection of study groups, comparability of groups, and ascertainment of either the exposure or the outcome. For case–control studies, the “Selection” domain (up to four stars) assesses the adequacy of case definition, representativeness of cases, selection of controls, and definition of controls. The “Comparability” domain (up to two stars) evaluates the comparability of cases and controls on the basis of the design or analysis. The “Exposure” domain (up to three stars) rates the method of exposure ascertainment, whether the same methods were used for cases and controls, and the non-response rate. For cross-sectional studies, the NOS criteria were adapted to evaluate the representativeness of the sample, sample size, non-respondents, assessment of exposure, and control for confounding factors. Studies with an NOS score of ≥ 7 were considered high quality and at low risk of bias (13).

### 2.5 Statistical analysis

All statistical analyses were performed using Stata software (version 16.0; StataCorp, College Station, TX). The adjusted OR was used as the primary effect measure across the included studies. Given that DR is a relatively common outcome, HRs and RRs were considered approximately equivalent to ORs; all effect estimates were pooled as ORs with 95% CIs. Heterogeneity was estimated using Cochran’s *Q* test and the *I*^2^ statistic. If the *I*^2^ value was ≤50% and the *Q*-test *p* > 0.10 (indicating low heterogeneity), a fixed-effects model was applied to determine the overall effect size. If *I*^2^ > 50% or the *Q*-test *p* ≤ 0.10 (indicating moderate or high heterogeneity), a random-effects model was used. To investigate the sources of heterogeneity, subgroup analyses were performed based on ethnicity, diabetes type, study design, and vitamin D assay method. Differences in effect sizes between subgroups were examined. Studies involving mixed diabetes-type populations were excluded from the diabetes-type subgroup analysis due to the inability to separate the data. Sensitivity analysis was conducted by sequentially removing one study at a time to evaluate the influence of each individual study on the overall result. Publication bias was assessed visually using Begg’s funnel plot and statistically using Egger’s linear regression test. A *p*-value of <0.05 in Egger’s test was considered suggestive of significant publication bias or small-study effects. If publication bias was detected, the non-parametric trim-and-fill method was applied to adjust the pooled estimate. All statistical tests were two-sided, and a *p*-value of <0.05 was considered statistically significant.

## 3 Results

### 3.1 Literature screening process

**Figure 1:**
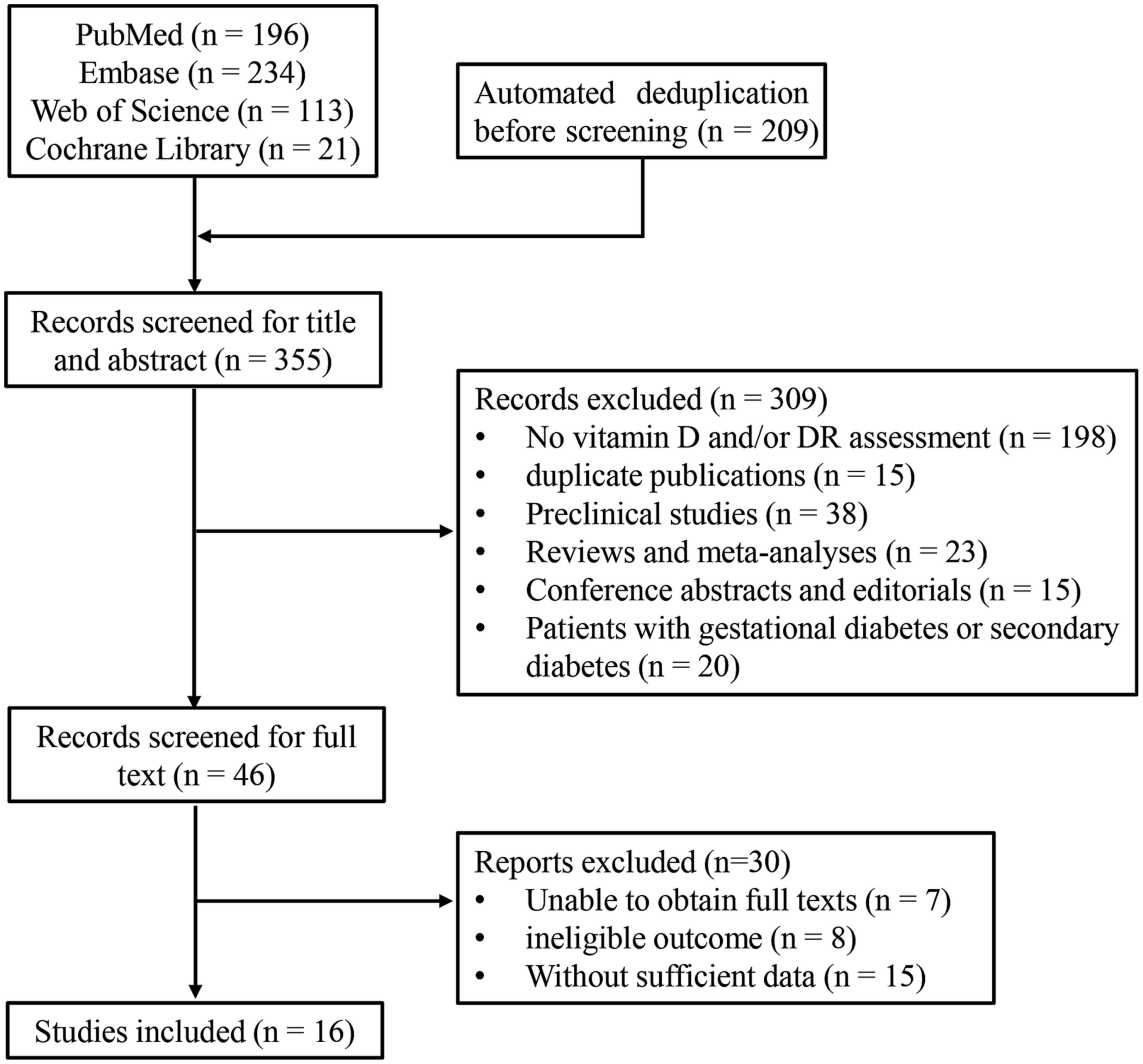
PRISMA 2020 flow diagram of the study selection process.

A total of 564 articles were identified from PubMed (*n* = 196), Embase (*n* = 234), the Web of Science (*n* = 113), and the Cochrane Library (*n* = 21). After automatically removing 209 duplicate records, 355 articles remained for title and abstract screening. Of these, 309 records were excluded for the following reasons: No vitamin D and/or DR assessment (*n* = 198), duplicate publications (*n* = 15), preclinical studies (*n* = 38), reviews and meta-analyses (*n* = 23), conference abstracts and editorials (*n* = 15), and patients with gestational diabetes or secondary diabetes (*n* = 20). Therefore, 46 articles remained for full-text screening. After excluding seven articles with unavailable full texts, eight articles with ineligible outcomes, and 15 articles lacking sufficient data, a total of 16 studies (8, 9, 14–27) were finally included in this meta-analysis (Figure 1).

### 3.2 Basic characteristics and quality assessment of enrolled studies

The key characteristics of the included studies are summarized in Table 1. These studies were published between 1 January 2015 and 24 February 2026 and collectively enrolled 19,987 diabetic patients from different geographical regions, including Asia (China, South Korea, India, and Saudi Arabia), Europe (Italy), and North America (United States). Among the participants, 3,638 were diagnosed with DR. Regarding study design, there were eight cross-sectional studies, seven case–control studies, and one cohort study. The serum 25(OH)D level was measured using standard techniques, with eight studies using chemiluminescent immunoassay (CLIA), six studies using liquid chromatography–tandem mass spectrometry (LC–MS/MS), and two studies using high-performance liquid chromatography (HPLC). The diagnosis of DR was based on established standards: 12 studies applied the Early Treatment Diabetic Retinopathy Study (ETDRS) grading system using fundus photography, three studies confirmed DR through ophthalmologist assessment, and one study used fluorescein angiography (FFA). Quality assessment using the NOS indicated that all studies were of high methodological quality, with scores ranging from 7 to 9 points.

**Table 1:** Baseline characteristics of the included studies.

First author (year)	Country	Study design	Diabetes type	Sample size	DR cases/total population	Vitamin D assay method	DR assessment method	Adjusted OR/HR (95% CI)	NOS score
Alam (9)	UK	Cross-sectional	T2DM	657	400/657	CLIA	ETDRS	1.25 (0.75–2.10)	7
Liu (14)	China	Case–control	T2DM	1,182	391/1,182	LC–MS/MS	FFA	2.10 (1.58–2.80)	7
Bajaj (15)	India	Cross-sectional	T2DM	736	251/736	CLIA	ETDRS	1.92 (1.40–2.64)	7
Park (16)	Korea	Case–control	T2DM	543	178/543	CLIA	ETDRS	2.35 (1.65–3.35)	8
Amer (17)	Italy	Cross-sectional	T1DM	312	98/312	HPLC	Ophthalmologist	2.18 (1.42–3.35)	7
Wang (18)	China	Case–control	T2DM	890	312/890	CLIA	ETDRS	1.89 (1.42–2.52)	8
Kaur (19)	India	Cross-sectional	T2DM	625	205/625	CLIA	ETDRS	1.75 (1.25–2.45)	7
Lee (20)	Korea	Case–control	T2DM	467	152/467	LC–MS/MS	ETDRS	2.41 (1.68–3.46)	8
Al-Daghri (21)	Saudi Arabia	Cross-sectional	T2DM	498	167/498	CLIA	ETDRS	1.68 (1.18–2.39)	7
Bian (22)	China	Case–control	T2DM	1,050	368/1,050	LC–MS/MS	ETDRS	2.05 (1.60–2.63)	9
Chaniyara (23)	India	Cross-sectional	Mixed	704	231/704	CLIA	ETDRS	1.80 (1.32–2.45)	7
Long (24)	China	Case–control	T1DM	285	95/285	LC–MS/MS	ETDRS	2.40 (1.55–3.72)	8
Morelli (25)	Italy	Cross-sectional	T1DM	198	62/198	HPLC	Ophthalmologist	2.05 (1.30–3.24)	7
Wong (26)	USA	Case–control	T2DM	610	201/610	CLIA	ETDRS	1.58 (1.12–2.23)	8
Zhao (27)	China	Cross-sectional	T2DM	579	190/579	LC–MS/MS	ETDRS	1.95 (1.42–2.68)	8
Hung (8)	China	Cohort	T2DM	10,651	337/10,651	LC–MS/MS	Ophthalmologist	1.45 (1.17–1.80)	7

T2DM, type 2 diabetes mellitus; T1DM, type 1 diabetes mellitus; DR, diabetic retinopathy; CLIA, chemiluminescent immunoassay; HPLC, high-performance liquid chromatography; LC–MS/MS, liquid chromatography–tandem mass spectrometry; ETDRS, Early Treatment Diabetic Retinopathy Study; OR, odds ratio; HR, hazard ratio; NOS, Newcastle–Ottawa Scale.

### 3.3 Meta-analysis results

**Figure 2:**
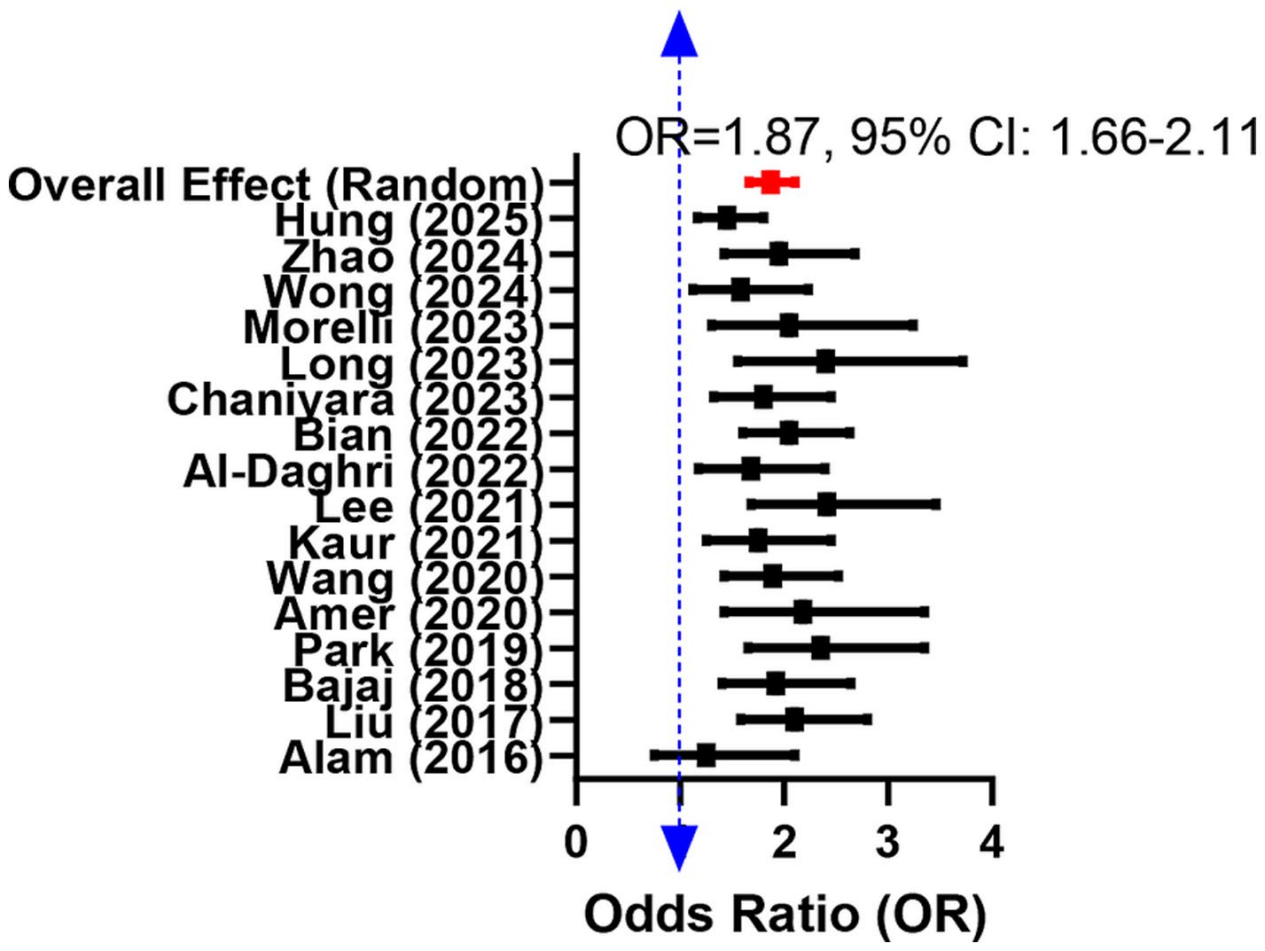
Forest plot of the meta-analysis results for the association between vitamin D levels and diabetic retinopathy. Pooled effect estimates were obtained from fully adjusted models.

The effect estimates from the 16 studies were pooled using a random effects model, and the results displayed a summary OR of 1.87 (95% CI: 1.66–2.11). This result showed a statistically significant 87% increase in the odds of DR among diabetic patients with the lowest serum vitamin D levels compared to those with the highest levels (Table 2). The forest plot visually presents the point estimate and confidence interval for each study, their respective weights in the meta-analysis, and the overall pooled estimate (Figure 2). Cochran’s *Q* test and the *I*^2^ statistic showed that there was a certain degree of heterogeneity among the results (*I*^2^ = 48.7%, *p* = 0.02).

**Table 2:** Study-specific and overall ORs for diabetic retinopathy comparing the lowest versus the highest serum vitamin D levels.

First author (year)	OR	95% CI (lower)	95% CI (upper)	Weight (%)
Overall effect (random)	1.87	1.66	2.11	100.0
Alam (9)	1.25	0.75	2.10	4.3
Liu (14)	2.10	1.58	2.80	7.2
Bajaj (15)	1.92	1.40	2.64	6.6
Park (16)	2.35	1.65	3.35	5.8
Amer (17)	2.18	1.42	3.35	4.8
Wang (18)	1.89	1.42	2.52	7.1
Kaur (19)	1.75	1.25	2.45	6.2
Lee (20)	2.41	1.68	3.46	5.7
Al-Daghri (21)	1.68	1.18	2.39	5.9
Bian (22)	2.05	1.60	2.63	7.9
Chaniyara (23)	1.80	1.32	2.45	6.6
Long (24)	2.40	1.55	3.72	4.6
Morelli (25)	2.05	1.30	3.24	4.5
Wong (26)	1.58	1.12	2.23	6.1
Zhao (27)	1.95	1.42	2.68	6.7
Hung (8)	1.45	1.17	1.80	9.0

OR, odds ratio; CI, confidence interval. Heterogeneity: *I*^2^ = 48.7%, *p* = 0.02.

### 3.4 Subgroup analysis

**Figure 3:**
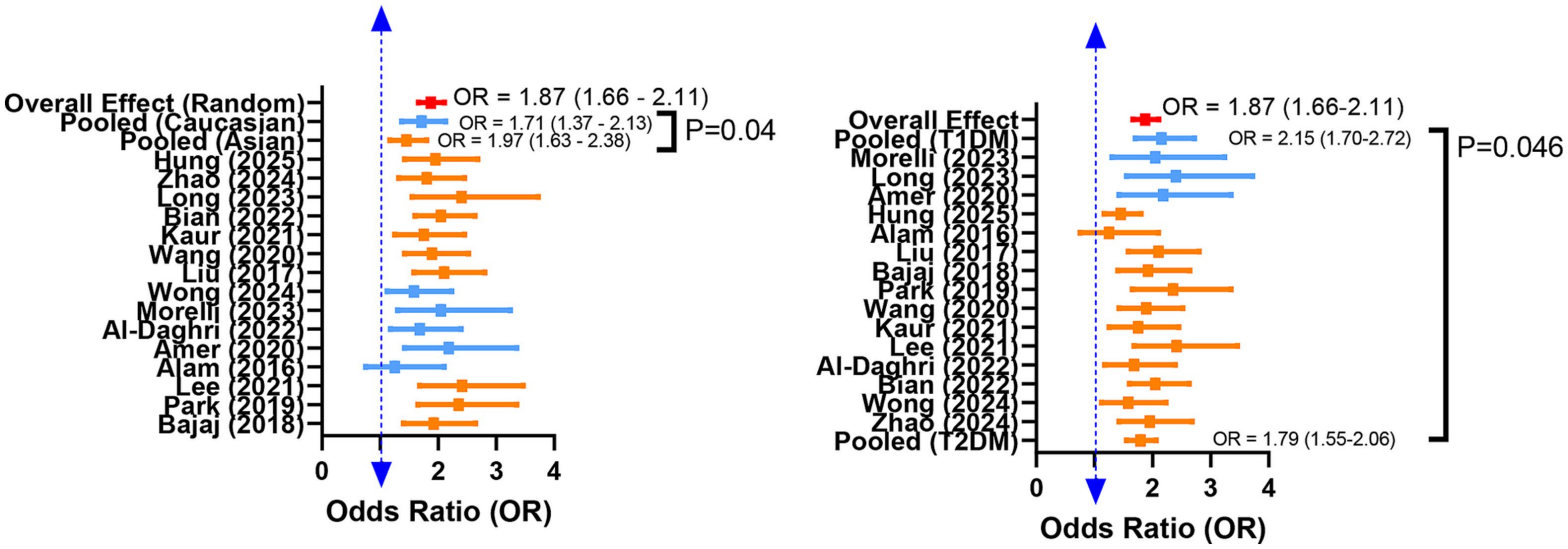
Forest plot of subgroup analysis results for the association between vitamin D levels and diabetic retinopathy, stratified by ethnicity and diabetes type.

To investigate the potential sources of heterogeneity, we performed subgroup analysis based on ethnicity, diabetes type, study design, and vitamin D assay (Table 3). By ethnicity, the pooled OR was higher in studies conducted in Asian populations (OR = 1.97, 95% CI: 1.63–2.38) than in those predominantly conducted in Caucasian populations (OR = 1.71, 95% CI: 1.37–2.13). The test for subgroup differences indicated a statistically significant effect modification by ethnicity (*p* = 0.040). When stratified by diabetes type, a stronger association was observed in T1DM patients (OR = 2.15, 95% CI: 1.70–2.72) than in those with T2DM (OR = 1.79, 95% CI: 1.55–2.06), with a significant between-group difference (*p* = 0.046). Statistical difference was not found in the effect of study design (*p* = 0.350) and vitamin D assay (*p* = 0.380) on this association. Forest plots stratified by ethnicity and diabetes type are presented in Figure 3. These subgroup findings suggest that Asian individuals and patients with T1DM may represent particularly high-risk populations for DR associated with vitamin D deficiency.

**Table 3:** Results of the subgroup analyses.

Subgroup	No. of studies	Pooled OR (95% CI)	*I* ^2^	*p* for interaction
Ethnicity				0.040
Asian	11	1.97 (1.63, 2.38)	51.2%	
Caucasian	5	1.71 (1.37, 2.13)	45.2%	
Diabetes type				0.046
Type 1	3	2.15 (1.70, 2.72)	0.0%	
Type 2	12	1.79 (1.55, 2.06)	52.4%	
Study design				0.350
Cross-sectional	8	1.74 (1.43, 2.12)	52.1%	
Case–control	7	1.99 (1.62, 2.44)	53.8%	
Vitamin D assay				0.380
Immunoassay	8	1.75 (1.46, 2.10)	53.4%	
Chromatography	8	1.98 (1.64, 2.39)	45.6%	

OR, odds ratio; CI, confidence interval. The *p* for interaction indicates whether the difference between subgroups was statistically significant.

### 3.5 Sensitivity analysis and publication bias

**Figure 4:**
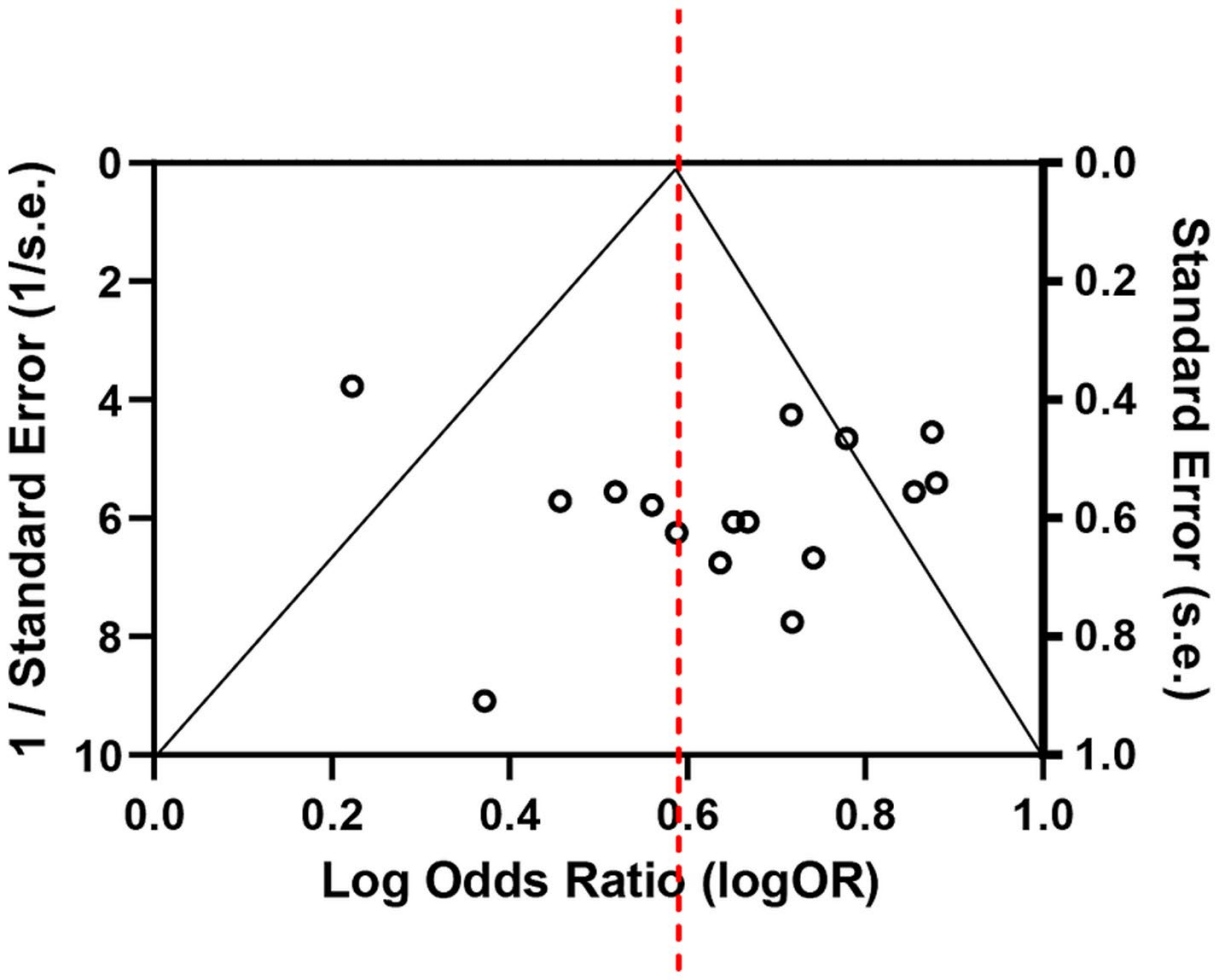
Begg’s funnel plot for the assessment of potential publication bias.

A leave-one-out sensitivity analysis was performed to evaluate the robustness of the primary meta-analysis results. Table 4 shows that the overall pooled effect was not significantly affected when omitting any single study, indicating that the results were robust. Publication bias was assessed using Begg’s funnel plot and Egger’s linear regression test. Begg’s funnel plot showed an approximately symmetrical distribution of effect sizes around the pooled estimate (Figure 4). Egger’s linear regression test demonstrated a non-significant intercept of 1.28 (*p* = 0.109), indicating no significant evidence of publication bias (Table 5).

**Table 4:** Results of the leave-one-out sensitivity analysis.

Omitted study	Pooled OR (95% CI)	*I*^2^ (%)
None	1.87 (1.66, 2.11)	48.7
Alam (9)	1.91 (1.70, 2.15)	47.1
Liu (14)	1.84 (1.63, 2.08)	48.3
Bajaj (15)	1.86 (1.65, 2.10)	50.0
Park (16)	1.83 (1.63, 2.06)	46.7
Amer (17)	1.85 (1.64, 2.09)	50.8
Wang (18)	1.86 (1.65, 2.10)	50.1
Kaur (19)	1.88 (1.66, 2.13)	49.7
Lee (20)	1.83 (1.63, 2.06)	46.2
Al-Daghri (21)	1.89 (1.67, 2.14)	49.2
Bian (22)	1.85 (1.64, 2.09)	50.6
Chaniyara (23)	1.88 (1.66, 2.13)	49.9
Long (24)	1.85 (1.64, 2.09)	51.0
Morelli (25)	1.86 (1.65, 2.10)	50.3
Wong (26)	1.90 (1.69, 2.14)	47.6
Zhao (27)	1.86 (1.65, 2.10)	50.2
Hung (8)	1.93 (1.72, 2.16)	43.8

OR, odds ratio; CI, confidence interval. The pooled estimate remained stable and statistically significant regardless of which single study was omitted, confirming the robustness of the main results.

**Table 5:** Results of Egger’s linear regression test for publication bias.

Coefficient	Value	Standard error	*t*-value	*p*-value
Intercept (bias)	1.28	0.75	1.71	0.109
Slope	0.59	0.14	4.21	< 0.001

Egger’s test assesses small-study effects. A non-significant intercept (*p* > 0.05) indicates no strong evidence of publication bias.

## 4 Discussion

The present meta-analysis synthesized evidence from 16 observational studies, demonstrating a significant inverse relationship between vitamin D levels and the odds of DR. Diabetic patients with low vitamin D status had an 87% higher odds of developing DR compared to those with sufficient levels. This association was found to be more pronounced in subgroups of Asian ethnicity and individuals with type 1 diabetes. These findings are biologically plausible, given the extensive physiological roles of vitamin D that intersect with key pathways implicated in DR pathogenesis.

The potential mechanisms underlying the association between vitamin D and DR are multifaceted. First, the well-established anti-inflammatory properties of vitamin D are of great significance. Vitamin D suppresses the activation of the NF-κB signaling pathway, thereby reducing the expression of key pro-inflammatory cytokines, including TNF-*α* and IL-6 (5). This may help ameliorate the chronic inflammatory microenvironment in the retina, a critical factor in blood-retinal barrier disruption and subsequent vascular leakage (28). Second, vitamin D exerts important antioxidant effects (29). It enhances the activity of antioxidant enzymes, such as SOD, thereby mitigating oxidative stress-induced damage to retinal pericytes and endothelial cells, which is a key feature of hyperglycemia-induced injury (29). Most directly, vitamin D acts as a negative regulator of VEGF, a central mediator of pathological angiogenesis and vascular permeability in DR. By inhibiting VEGF expression and signaling, potentially through the modulation of hypoxia-inducible factor-1α (HIF-1α), vitamin D directly counteracts a fundamental process in DR progression (30, 31). Furthermore, its inhibitory effect on the RAS contributes to improved endothelial function and reduced tissue fibrosis (32). These anti-inflammatory, antioxidant, and anti-angiogenic mechanisms provide a compelling scientific basis for the observed association. Our findings support the notion that vitamin D deficiency is linked to adverse diabetic microvascular complications, consistent with recent expert consensus highlighting the importance of vitamin D in risk stratification and preventive strategies for cardiovascular diseases (33).

Subgroup analyses indicated that ethnicity and diabetes type may be the potential effect modifiers and sources of heterogeneity. This meta-analysis found a stronger association between vitamin D and DR in Asian populations than in predominantly Caucasian groups. This finding may be attributable to several factors. First, the prevalence of vitamin D deficiency is higher in many Asian countries, influenced by factors such as skin pigmentation, cultural practices limiting sun exposure, and dietary habits with lower intake of fortified dairy products (34). This may result in a population-level susceptibility. Second, genetic differences between Asian and Caucasian populations, including variations in the prevalence of vitamin D receptor (VDR) gene polymorphisms, may modulate individual responsiveness to vitamin D (35). We also found that the association between vitamin D and DR was stronger in patients with type 1 diabetes. This may be explained by the early onset and lifelong disease course of T1DM, leading to prolonged exposure to potential vitamin D insufficiency (36). In addition, the autoimmune nature of T1DM may interact more directly with the immunomodulatory effects of vitamin D (37). These subgroup findings highlight the clinical importance of prioritizing vitamin D assessment and management in diabetic patients of Asian descent or with T1DM.

This meta-analysis has several notable strengths. First, subgroup analyses were performed to explore potential effect modifiers, such as ethnicity and diabetes type, which may provide valuable insights into the specificity of the association across different populations. Second, we extracted adjusted effect estimates from the included studies whenever possible, which may reduce the impact of potential confounding factors and improve the reliability of the pooled results. This meta-analysis also has certain limitations that should be acknowledged. First, although we included adjusted effect estimates, there are still important sources of residual confounding, such as physical activity, sunlight exposure, nutritional status, renal function severity, and socioeconomic status, which may affect the observed association. Second, there is a possibility of reverse causality. Patients with severe DR may reduce outdoor activity and sunlight exposure, thereby leading to lower vitamin D levels. Third, variations in the definition of vitamin D deficiency (different cutoff values) and laboratory assay methods across the included studies may introduce measurement bias and contribute to statistical heterogeneity, thereby influencing the pooled effect estimates. Fourth, all included studies were observational; therefore, definitive causal conclusions regarding the relationship between vitamin D deficiency and DR cannot be drawn. Future prospective cohort studies and randomized controlled trials are needed to clarify the causal association between vitamin D status and the risk of DR.

## 5 Conclusion

In conclusion, this meta-analysis provides robust evidence for the association between low serum vitamin D levels and increased odds of DR, and this association was more significant in Asian populations and T1DM patients. These results suggest that routine monitoring of serum vitamin D levels should be considered in the management of DR, especially for high-risk populations. Future research should prioritize large-scale prospective cohort studies to better explore the temporal and potential causality between vitamin D status and DR incidence. Furthermore, well-designed randomized controlled trials are essential to determine the efficacy, optimal dosage, and target serum levels of vitamin D supplementation for the primary prevention of DR in diabetic populations.

## Data Availability

The original contributions presented in the study are included in the article/Supplementary material, further inquiries can be directed to the corresponding author.
