# Supplementary material for: Association between serum vitamin D levels and the risk of diabetic retinopathy: a meta-analysis
Source: Front Med (Lausanne). 2026 Apr 1;13:1771156. doi: 10.3389/fmed.2026.1771156 (PMC13079128; doi:10.3389/fmed.2026.1771156)
Supplement: Supplementary file 1 [file Table_1.docx]

Search terms comprised “Vitamin D” OR “Cholecalciferol” OR “Ergocalciferols” OR “25-Hydroxyvitamin D 2” OR “Hydroxycholecalciferols” OR “vitamin D” OR “cholecalciferol” OR “ergocalciferol” OR “calcifediol” OR “25-hydroxyvitamin D” OR “25(OH)D” AND “Diabetic Retinopathy” OR “Diabetes Complications” OR “diabetic retinopathy” OR “diabetic retinopathies” OR “DR” OR “nonproliferative diabetic retinopathy” OR “NPDR” OR “proliferative diabetic retinopathy” OR “PDR”.
